# Methylprednisolone is related to lower incidence of postoperative bleeding after flow diverter treatment for unruptured intracranial aneurysm

**DOI:** 10.3389/fnagi.2023.1029515

**Published:** 2023-04-18

**Authors:** Linggen Dong, Qingyuan Liu, Xiheng Chen, Longhui Zhang, Jiejun Wang, Qichen Peng, Jiangan Li, Hongwei He, Peng Liu, Ming Lv

**Affiliations:** ^1^Department of Neurosurgery Center, Beijing Tiantan Hospital, Capital Medical University, Beijing, China; ^2^Beijing Neurosurgical Institute, Capital Medical University, Beijing, China; ^3^Department of Emergency, The Affiliated Wuxi No. 2 People’s Hospital of Nanjing Medical University, Wuxi, Jiangsu, China

**Keywords:** unruptured intracranial aneurysm, flow diverter, methylprednisolone, postoperative bleeding, endovascular treatment

## Abstract

**Background and objectives:**

Regarding the anti-inflammatory effect, methylprednisolone is a candidate to prevent patients with unruptured intracranial aneurysms (UIAs) from postoperative bleeding (PB) after flow diverter (FD) treatment. This study aimed to investigate whether methylprednisolone is related to a lower incidence of PB after FD treatment for UIAs.

**Methods:**

This study retrospectively reviewed UIA patients receiving FD treatment between October 2015 and July 2021. All patients were observed until 72  h after FD treatment. The patients receiving methylprednisolone (80  mg, bid, for at least 24 h) were considered as standard methylprednisolone treatment (SMT) users, otherwise as non-SMT users. The primary endpoint indicated the occurrence of PB, including subarachnoid hemorrhage, intracerebral hemorrhage, and ventricular bleeding, within 72 h after FD treatment. This study compared the incidence of PB between SMT users and non-SMT users and investigated the protective effect of SMT on PB after FD treatment using the Cox regression model. Finally, after controlling the potential factors related to PB, we performed subgroup analysis to further confirm the protective effect of SMT on PB.

**Results:**

This study finally included 262 UIA patients receiving FD treatment. PB occurred in 11 patients (4.2%), and 116 patients (44.3%) received SMT postoperatively. The median time from the end of surgery to PB was 12.3 h (range: 0.5–48.0 h). SMT users had a lower incidence of PB comparing with non-SMT users (1/116, 0.9% vs. 10/146, 6.8%, respectively; *p* = 0.017). The multivariate Cox analysis demonstrated that SMT users (HR, 0.12 [95%CI, 0.02–0.94], *p* = 0.044) had a lower risk of PB postoperatively. After controlling the potential factors related to PB (i.e., gender, irregular shape, surgical methods [FD and FD + coil] and UIA sizes), the patients receiving SMT still had a lower cumulative incidence of PB, comparing with patients receiving non-SMT (all *p* < 0.05).

**Conclusion:**

SMT was correlated with the lower incidence of PB for patients receiving FD treatment and may be a potential method to prevent PB after the FD treatment.

## Introduction

Intracranial aneurysms are the leading cause of nontraumatic subarachnoid hemorrhage, and are found in 4% of the American population (Connolly et al., 2012) and in 7% of the Chinese population (Li et al., 2013). More than 50% of intracranial aneurysms are unruptured intracranial aneurysm (UIAs), which are asymptomatic and found incidentally (Tawk et al., 2021). The incidence of intracranial aneurysms increases with age, so middle-aged and elderly patients account for a large proportion (Smith et al., 2015). Notably, aging patients also have a high incidence of treatment complications and a relatively poor prognosis due to poor physical function and multiple concomitant diseases, which makes patients unable to benefit from surgical treatment (Brinjikji et al., 2013a,b). With the development of neurointerventional devices and techniques, endovascular treatment is safer and more effective, and has gradually become the main treatment modality for intracranial aneurysms. Flow diverter (FD) is one of the effective methods to treat UIAs. (Becske et al., 2013; Brinjikji et al., 2013a,b; Kallmes et al., 2015, 2016; Akinduro et al., 2020; Hanel et al., 2020; Sweid et al., 2020) Postoperative bleeding (PB) is a life-threatening event after the FD treatment. The previous studies reported that PB occurred in approximately 4% of UIA patients receiving FD treatment (Arrese et al., 2013; Brinjikji et al., 2013a,b; Rouchaud et al., 2016; Kang et al., 2021). Given the high mortality and high risk of poor outcomes caused by PB after FD treatment, it is crucial to investigate the prophylactic methods to prevent UIA patients from PB.

Acute inflammation response and thrombus formation after FD treatment was recognized as the one of potential mechanisms of PB (Chow et al., 2012; Hampton et al., 2018). On this basis, some researchers believed that anti-inflammatory treatment, such as methylprednisolone administration, would help prevent patients from PB after FD treatment (Ikeda et al., 2015; Hampton et al., 2018). However, these studies involved small samples and had no standard medication use, limiting the utility of these conclusions.

Here, a study was conducted to retrospectively review the UIA patients receiving FD treatment in our institution. The factors related to PB were investigated. This study aimed to verify whether methylprednisolone was correlated with the lower incidence rate of PB.

## Methods

### Study population

The UIA patients receiving neurointerventional treatment between October 2015 and July 2021 were retrospectively reviewed. Patients were selected according to the following criteria: (1) 18–75 years, (2) no history of aneurysmal subarachnoid hemorrhage, and (3) the UIAs were treated by FD or FD-based method (FD plus coils). Besides, the patients had the following situations were excluded: (1) with cerebrovascular malformations or intracranial tumors, such as brain arteriovenous malformation or glioma, (2) with traumatic, bacterial, or atrium myxomas aneurysms, (3) suffering from multiple intracranial aneurysms, (4) suffering from intraoperative aneurysm rupture, (5) suffering from intraoperative thrombotic events and needed tirofiban treatment, and (6) unable to track the detail of postoperative medication using or with a non-standard medication using postoperatively.

### Clinical information and aneurysm morphology

The demographic information (age, gender, history of smoking, and alcohol consumption) and comorbidities (hypertension, dyslipidemia, diabetes mellitus, and coronary artery disease) were collected based on the electronic medical records. In this study, the postoperative administration was recorded, mainly including the methylprednisolone. The dosage, frequency, and duration were also recorded.

The aneurysm morphology was measured with the 3-dimensional digital subtraction angiography. Two investigators (investigator 1 and investigator 2) performed the measurement. The discrepancy was managed by consulting a senior neuro interventionist.

The location, aneurysm size, bifurcation, and irregular shape were measured and recorded by each investigator. An irregular shape was defined as a small bleb(s) or secondary aneurysm(s) protruding from the aneurysm fundus or bi−/multi-lobular aneurysm fundus. The aneurysm size was categorized into three groups: <7 mm, 7–10 mm, and >10 mm.

### Postoperative management

In our institution, all patients received standard care, including blood pressure management and dual antiplatelet treatment. After the FD treatment, lowering the systolic pressure to 120–140 mmHg was the target. Patients received a dose of low molecular weight heparin after the surgery, and the dual antiplatelet therapy (aspirin 100 mg plus clopidogrel 75 mg) was maintained for 6 weeks postoperatively, and aspirin (100 mg) monotherapy continued for 6 months. Currently, there was no consensus or guideline on the usage of methylprednisolone. Thus, the usage of methylprednisolone was based on the experiences of senior neuro interventionists. Based on this fact, we defined patients receiving standard methylprednisolone (80 mg, bid, for at least 1 day (24 h), but usually no more than 3 days) as standard methylprednisolone treatment (SMT) group, otherwise as non-SMT group.

### Identification of primary outcome

The primary outcome in this study was postoperative bleeding (PB). All patients routinely underwent computed tomography (CT) scans at 4 h and 24 h postoperatively. In addition, once the state of consciousness deteriorated, a CT would be conducted immediately. We identified the PB when patients (1) had decreased consciousness or positive signs of the nervous system, and the patient’s Glasgow Coma Scale score less than 15 and (2) had a new intracranial hemorrhage on postoperative CT. The PB events, includes subarachnoid hemorrhage, intracerebral hemorrhage, and ventricular bleeding, which were related to FD treatment. Based on the occurrence of PB, the included patients were categorized into two groups: PB users and non-PB users. The interval from the end of surgery to PB or 72 h after surgery was regarded as the observation time.

### Statistical analysis

The statistical analyses were conducted by SPSS 24.0 (SPSS, Chicago, IL). The results suggested two-sided *p* < 0.05, which indicated statistical significance. Continuous variables with normal distribution were expressed as means and standard deviation (SD), and medians and interquartile range if otherwise. Categorical variables were expressed as numbers (no.) and percentages (%). Besides, the differences between PB and non-PB groups in continuous variables were compared by performing student’s t-tests or Wilcoxon rank-sum tests; the differences in categorical variables were compared with chi-square tests or Fisher’s exact tests. The survival analysis was conducted using the Kaplan–Meier method. Then, the parameters with *p* < 0.1 in univariate Cox regression analysis were input into a multivariate Cox model. The crude HR of multivariate Cox model was further adjusted using the factors related to PB (locations, aneurysm size, shape and surgical methods). The results were presented as hazard ratio (HR) and 95% confidence interval (CI). To further investigate whether SMT is related to low incidence of PB, we further performed subgroup analysis based on factors, including locations, aneurysm size, shape and surgical methods. The incidence of PB and its 95% CI were calculated.

## Results

### Baseline information and aneurysm characteristics

**Figure 1:**
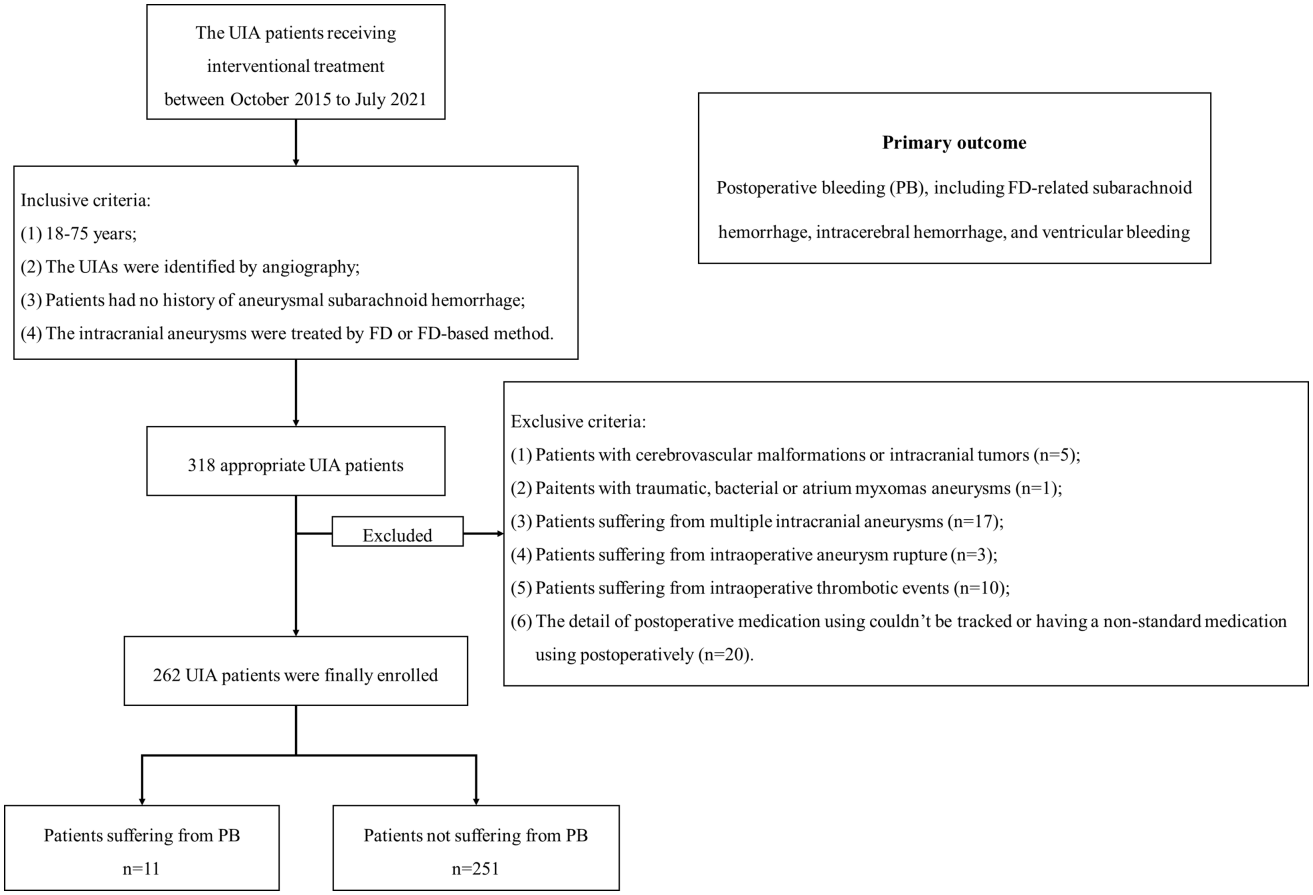
The flow chart of patient enrollment. We included 262 patients with 262 UIAs in this study. Among them, 11 patients suffered from PB. UIA, unruptured intracranial aneurysm; PB, postoperative bleeding.

The flow chart was presented in Figure 1. This study finally included 262 patients, with 205 female (78.2%) and an age ranging from 18 to 75. Among them, PB occurred in 11 patients (4.2%) after FD treatment, and 116 patients (44.3%) received SMT postoperatively. The baseline information grouped by PB was summarized in Table 1. 119 patients (45.4%) received FD treatment, and 143 patients (54.6%) received FD + coils treatment. The UIA size of 89 (34.3%) as <7 mm, 51 (19.5%) as 7–10 mm and 121 (46.2%) as >10 mm.

**Table 1:** The baseline information of patients grouped by PB.

Characteristics	PB *n* = 11	non-PB *n* = 251	*p* value
Age - mean ± SD - years	57.7 ± 8.0	53.3 ± 10.2	0.203
Male-no. (%)	11 (100.0%)	194 (77.3%)	0.077
Comorbidities-no. (%)			
Hypertension	5 (23.5%)	88 (39.1%)	0.482
Dyslipidemia	2 (18.2%)	90 (35.9%)	0.230
Diabetes mellitus	0 (0.0%)	15 (6.0%)	0.405
Coronary artery disease	0 (0.0%)	14 (5.6%)	0.422
Current smoker-no. (%)	0 (0.0%)	28 (11.2%)	0.242
Regular alcohol abuse-no. (%)	0 (0.0%)	21 (8.4%)	0.318
Surgical methods			0.216
FD	7 (63.6%)	112 (44.6%)	
FD + coils	4 (36.4%)	139 (55.4%)	
Postoperative SMT			0.017^a^
Yes	1 (9.1%)	115 (45.8%)	
No	10 (90.9%)	136 (54.2%)	
Location-no. (%)			0.361
ICA	9 (81.8%)	226 (90.0%)	
MCA	0 (0.0%)	5 (2.0%)	
ACA/AComA/PC	2 (18.2%)	20 (8.0%)	
Bifurcation-no. (%)	2 (18.2%)	14 (5.6%)	0.088
Irregular shape-no. (%)	2 (18.2%)	36 (14.3%)	0.724
Aneurysm size-no. (%)			0.575
<7 mm	3 (27.3%)	86 (34.3%)	
7-10 mm	2 (18.2%)	49 (19.5%)	
>10 mm	6 (54.5%)	116 (46.2%)	

a The parameter was significant.

PB, postoperative bleeding; FD, flow diverter; SMT, standard methylprednisolone treatment; ICA, internal carotid artery; MCA, middle cerebral artery; ACA, anterior cerebral artery; AComA, anterior communicating artery; PC, posterior circulation.

The details of patients suffering from PB was given in Supplementary Table S1. The SMT users had a lower PB rate comparing with non-SMT users (1/115, 0.9% vs. 10/146, 6.8%, respectively; *p* = 0.017). There was no statistical significance in age, gender, hypertension, dyslipidemia, diabetes mellitus, coronary artery disease, current smoker, regular alcohol abuse, surgical methods, location, bifurcation, irregular shape and aneurysm size was also not discovered between PB and non-PB users (all *p* > 0.05).

### Postoperative SMT is related to lower risk of PB

**Figure 2:**
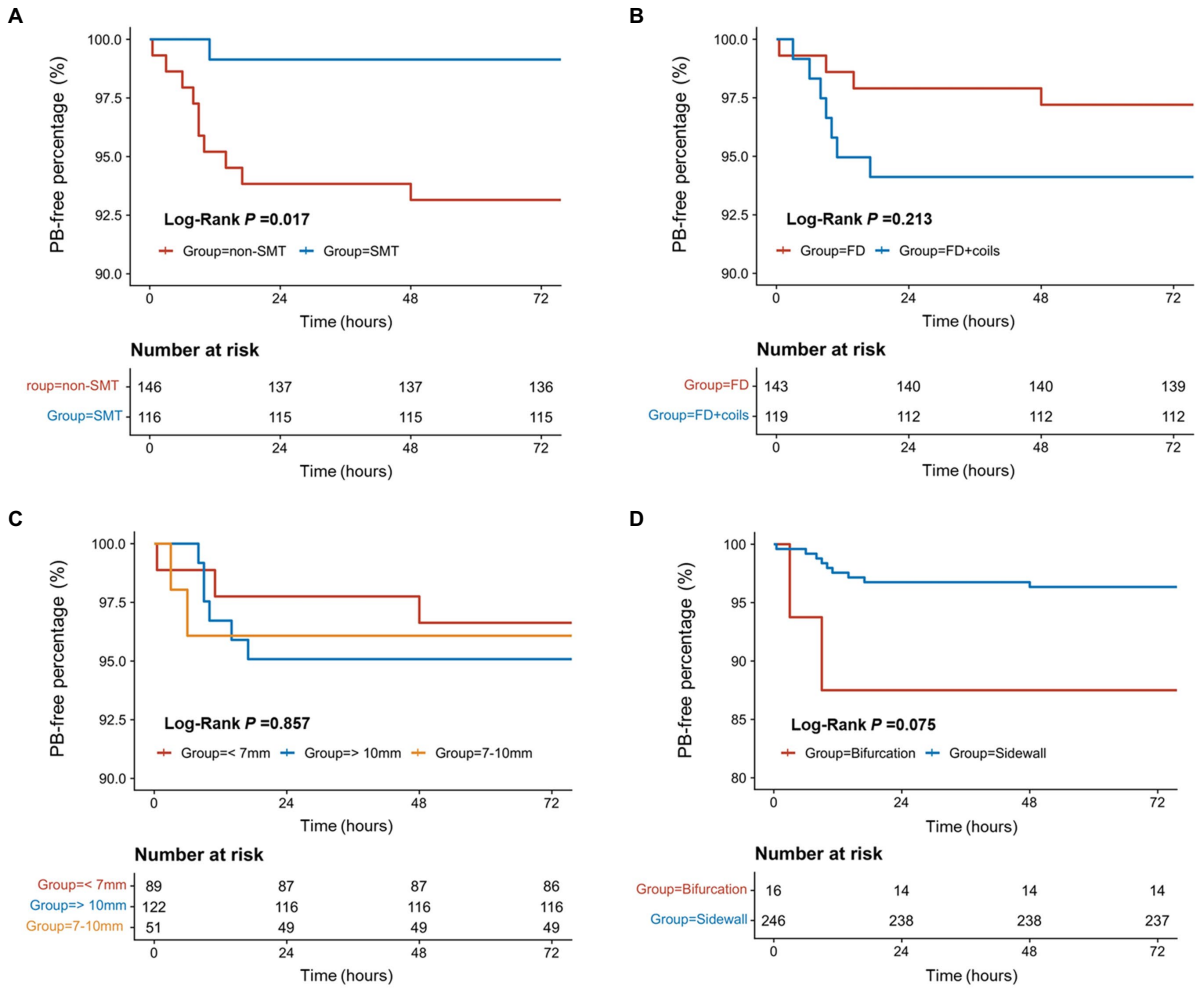
The survival analysis for the factors related to postoperative bleeding. **(A)** The survival analysis of SMT/non-SMT for PB. **(B)** The survival analysis of surgical methods for PB. **(C)** The survival analysis of different aneurysm sizes for PB. **(D)** The survival analysis of bifurcation/sidewall for PB. PB, postoperative bleeding; SMT, standard methylprednisolone treatment; FD, flow diverter.

The medium time from the end of surgery to PB was 12.3 h (range: 0.5–48.0 h). The survival analysis showed that the patients receiving SMT postoperatively had a lower incidence of PB (Figure 2A, *p* = 0.017). As shown in Figures 2B–D and Table 2, the incidence of PB had no significance between patients receiving FD and FD + coils (*p* = 0.213), or different aneurysm size (*p* = 0.857), or bifurcation and sidewall (*p* = 0.075).

**Table 2:** The survival analysis for the factors related to PB.

Characteristics	n^a^/N	*p* value
Age > 60 years	4/80	0.829
Male	11/205	0.701
Hypertension	4/120	0.202
Dyslipidemia	4/107	0.357
Diabetes mellitus	0/22	0.251
Coronary artery disease	1/21	0.876
Current smoker	3/40	0.533
Regular alcohol abuse	0/28	0.190
Surgical methods		0.213
FD	7/119	
FD + coils	4/143	
SMT users	1/116	0.017^b^
Location		0.860
ICA	15/276	
MCA	0/5	
AcomA/ACA/Posterior	2/33	
Bifurcation	8/20	<0.001^b^
Irregular shape	4/47	0.281
Aneurysm size		0.348
<7 mm	8/116	
7-10 mm	1/59	
>10 mm	8/139	

a The accumulative number of PB.

b The parameter was significant.

PB, postoperative bleeding; ACA, anterior cerebral artery; AcomA, anterior communicating artery; ICA, internal carotid artery; MCA, middle cerebral artery.

**Figure 3:**
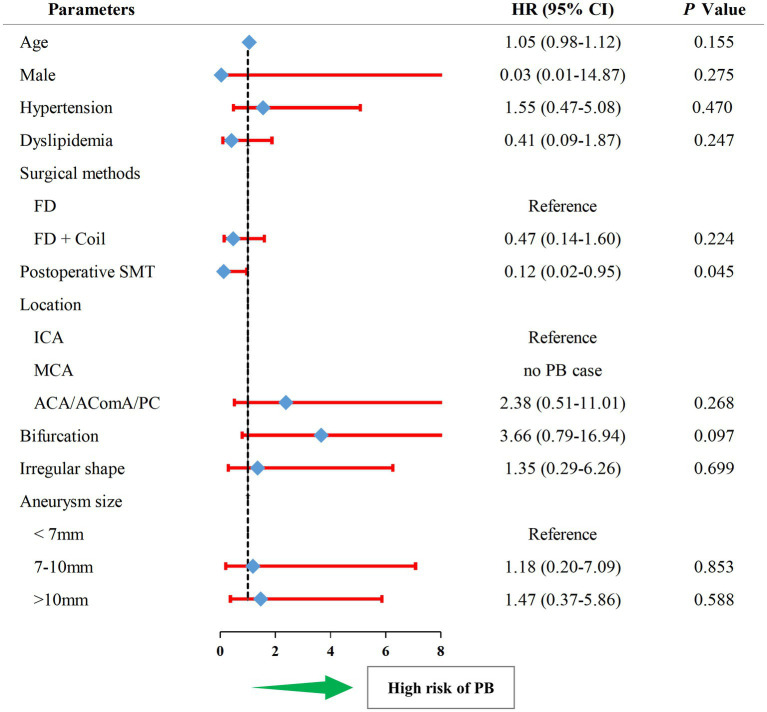
The forest plot of univariate Cox regression analysis for risk factors related to postoperative bleeding. HR, hazard ratio; PB, postoperative bleeding; SMT, standard methylprednisolone treatment; ACA, anterior cerebral artery; AComA, anterior communicating artery; PC, posterior circulation; FD, flow diverter.

The univariate Cox analysis revealed SMT (HR, 0.12; 95%CI, 0.02–0.95; *p* = 0.045) as the protective factor for PB (Figure 3, and also see in Supplementary Table S2). Consequently, the parameters, including SMT and bifurcation, were input into a multivariate Cox model. As showed in Table 3, SMT (HR, 0.12; 95%CI, 0.02–0.94; *p* = 0.044) was related to lower risk of PB independently. Furthermore, after being adjusted by irregular shape, UIA location, and UIA size, the conclusion was consistent (HR, 0.10; 95%CI, 0.01–0.84; *p* = 0.034).

**Table 3:** Multivariate Cox analysis^a^ for risk factors related to PB.

Parameters	Crude		Adjusted^b^	
	HRs	*p* value	HRs	*p* value
Postoperative SMT	0.12 (0.02–0.94)	0.044	0.10 (0.01–0.84)	0.034
Bifurcation	3.81 (0.82–17.66)	0.087		

a The result was presented as HR and 95% confidence interval.

b The result was adjusted by gender, irregular shape, location, aneurysm size and surgical methods.

PB, postoperative bleeding; HR, hazard ratio.

### The incidence of PB in different groups

**Figure 4:**
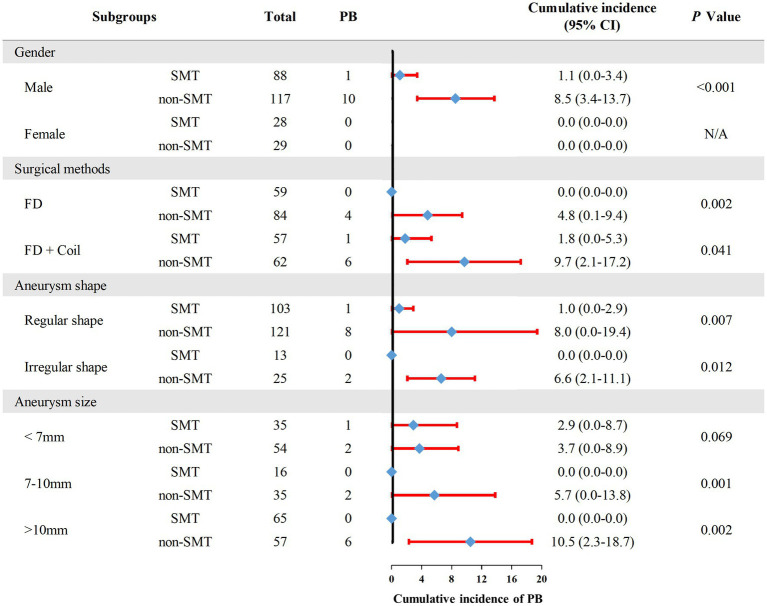
The incidence of postoperative bleeding in each subgroup. PB, postoperative bleeding; SMT, standard methylprednisolone treatment; FD, flow diverter.

To further investigate whether SMT is related to lower incidence of PB, we performed subgroup analysis (Figure 4). The result showed that for different gender, patients receiving different surgical methos, and patients with different UIA sizes or different UIA shape, SMT users still had a lower incidence of PB, with an exception to patients with UIA size as <7 mm.

## Discussion

In this current study, for patients receiving FD treatment, the multivariate COX analysis showed SMT postoperatively was the independent risk factor related to lower risk of PB. After controlling the factors related to PB, we confirmed the patients receiving SMT postoperatively still had a lower risk of PB, which suggested the prophylactic role of methylprednisolone for PB after the FD treatment.

There has been a growing number of studies demonstrating inflammation plays a crucial role in the structural deterioration and subsequent rupture of the intracranial aneurysm wall (Chow et al., 2012; Chalouhi et al., 2013; Hosaka and Hoh, 2014). Inflammation can directly cause intracranial aneurysm wall degradation, thereby leading to aneurysmal enlargement (Aoki and Nishimura, 2010). Under the effect of inflammation, the degenerated aneurysmal wall became too fragile to resist the hemodynamic stress, and finally ruptured (Kataoka, 2015). At present, the drug therapy that can prevent the progression and rupture of intracranial aneurysms are still under investigation, several studies reported that methylprednisolone might be able to improve clinical outcome (Gomis et al., 2010; Chow et al., 2012; Ikeda et al., 2015). Methylprednisolone is a synthetic glucocorticoid, primarily known for its anti-inflammatory and immunosuppressive effects, and achieves this effect by regulating the number and function of leukocytes, cytokines and chemokines (Xavier et al., 2016; Timmermans et al., 2019). In addition, it also suppress cyclooxygenase expression, thus potentiating the anti-inflammatory effect (Jun et al., 1999). Therefore, methylprednisolone may be a suitable candidate to prevent UIA from PB after the FD treatment, and a SMT (80 mg, bid) was recommended after FD treatment to prevent patients from PB. However, our conclusion needs to be confirmed by randomized controlled trials or case–control studies.

The concept of treating UIAs using FD is closely related to hemodynamic basis of aneurysms. Many problems about FD treatment were investigated using hemodynamic methods, including PB after FD treatment. Brunozzi et al. reported that the mean ipsilateral middle cerebral artery flow velocity was increased significantly in patients suffering from delayed ipsilateral parenchymal hemorrhage (Brunozzi et al., 2018). Similar results were also reported by Li et al., that the mean velocity of arteries related to delayed ipsilateral parenchymal hemorrhage was increased and the imbalance in blood flow distribution of distal arteries might play an important role, after FD treatment for UIAs (Li et al., 2021). Li et al. showed that an unstable flow pattern and high energy loss within released FD may be important hemodynamic risk factors related to PB (Li et al., 2019). Cebral et al. also reported that high pressure within aneurysms and continued inflow into the aneurysms were potential mechanisms of PB after FD treatment (Cebral et al., 2011). Notably, these abnormal hemodynamic conditions usually lead to an inflammation infiltration in the aneurysm wall (Meng et al., 2014; Tulamo et al., 2018), which may be inhibited by methylprednisolone. However, *in vitro* and *in vivo* studies were still needed to confirm this finding.

UIA size is reported as a factor related to PB. Sweid et al. reported that a total of 88% of aneurysm rupture cases occurred during the first month, and 55% were large aneurysms measuring at least 20 mm (Sweid et al., 2020). Similarly, Rouchaud et al. reported that 76.6% of aneurysm rupture occurred within one month of the procedure, and about 50% of which involved giant aneurysms (Rouchaud et al., 2016). In addition, the PLUS study in China demonstrated that large aneurysm size, especially sizes >10 mm, was an independent predictor of aneurysm rupture in the early postoperative period (Kang et al., 2021). However, our study did not reach the above conclusion, we suppose the possible reasons for this are limited patients suffering from PB. Thus, the patients with UIAs >10 mm had the highest rate of PB comparing with patients with UIAs <10 mm, whereas the difference was not significant. In further subgroup analysis, we found that the larger the aneurysm, the more effective the use of methylprednisolone is in preventing PB. Thus, we recommend that SMT can be used routinely for patients with large UIAs (aneurysm size >10 mm).

Several studies recommended that giant aneurysms are treated with FD + coils in order to protect the dome of the aneurysm and prevent PB (Turowski et al., 2011; Berge et al., 2012; Velioglu et al., 2012). Indeed, our study found that patients receiving FD + coils had a lower rate of PB comparing with patients receiving only FD, suggesting that the high-density packing would be more protective against PB. In subsequent subgroup analysis, our data showed that SMT users had a lower incidence of PB in patients receiving only FD or FD + coils. Thus, SMT is suitable potential method to prevent UIA patients from PB.

## Limitations

There are some limitations to this study. First, our study was a single-center, retrospective study. Consequently, patient selection and administration bias would limit our conclusion. Second, this study only included patients receiving SMT (80 mg, bid) while neglecting the effect of different doses and the duration of methylprednisolone administration on the risk of PB after the FD treatment. Third, this study did not consider the effect from neuro interventionists’ option on giving SMT after the FD treatment; furthermore, the administration of methylprednisolone is based on the experience of senior neuro interventionist. Whether SMT can actually protect UIA patients from PB still needs randomized controlled trials to confirm. Fourth, there may be potential risk factors related to the PB after FD treatment, such as aneurysm size and irregular shapes, which were reported in previous studies (Sweid et al., 2020; Kang et al., 2021). Fifth, we did not perform platelet function tests on every patient admitted to the hospital, which is one of the shortcomings of this study, and we will add this part in the follow-up study. Sixth, only Chinese patients were included in this study, restricting the generality of our conclusion. Although aforementioned limitations existed, this study revealed the protective role of methylprednisolone for PB after the FD treatment and provided a new idea for the clinical prevention of PB after the FD treatment.

## Conclusion

SMT was correlated with the lower incidence of PB for patients receiving FD treatment and may be a potential method to prevent PB after the FD treatment. For patients with large UIAs (aneurysm size >10 mm), we recommended administration of SMT routinely. Further randomized controlled trials or case–control studies were still needed to confirm our conclusion.

## Data availability statement

The raw data supporting the conclusions of this article will be made available by the authors, without undue reservation.

## Ethics statement

This study was reviewed and approved by the Ethics Committee of Beijing Tiantan Hospital. Written informed consent to participate in this study was provided by the patients or their legal guardian/next of kin.

## Author contributions

LD and QL: conception and design. LD, XC, LZ, JW, and QP: acquisition of data. QL: analysis and interpretation of data. LD and QL: drafting the article. PL, JL, and HH: critically revising the article. LD, QL, XC, LZ, JW, QP, JL, HH, PL, and ML: reviewing submitted version of manuscript. ML: approving the final version of the manuscript on behalf of all authors and study supervision. All authors contributed to the article and approved the submitted version.

## Funding

This study was supported by the National Natural Science Foundation of China (grant nos. 82271319 and 81901197), “National Key Research and Development Program of the 14th Five-Year Plan (grant no. 2021YFC2501100),” “Wuxi Taihu Lake Talent Plan, Leading Talents in Medical and Health Profession (grant no. 202014)” and “Wuxi Taihu Lake Talent Plan, Team in Medical and Health Profession (grant no. TH202109).”

## Conflict of interest

The authors declare that the research was conducted in the absence of any commercial or financial relationships that could be construed as a potential conflict of interest.

## Publisher’s note

All claims expressed in this article are solely those of the authors and do not necessarily represent those of their affiliated organizations, or those of the publisher, the editors and the reviewers. Any product that may be evaluated in this article, or claim that may be made by its manufacturer, is not guaranteed or endorsed by the publisher.

## Abbreviations

PB: Postoperative bleeding
FDF: low diverter
UIA: Unruptured intracranial aneurysm
ACA: Anterior cerebral artery
AComA: Anterior communicating artery
ICA: Internal carotid artery
MCA: Middle cerebral artery
