# Supplementary material for: Methylprednisolone is related to lower incidence of postoperative bleeding after flow diverter treatment for unruptured intracranial aneurysm
Source: Front Aging Neurosci. 2023 Apr 18;15:1029515. doi: 10.3389/fnagi.2023.1029515 (PMC10151685; doi:10.3389/fnagi.2023.1029515)
Supplement: Supplementary file 1 [file Table_1.DOCX]

Supplementary materials to *Methylprednisolone is related to lower incidence of postoperative bleeding after flow diverter treatment for unruptured intracranial aneurysm*

**Supplementary tables**

**Supplemental table 1. Clinical details in 11 patients who experienced postoperative bleeding after flow diverter treatment.**

| No. | Age (years)/gender | Symptoms at admission | Aneurysm location | Postoperative bleeding | Hematoma volume (ml) | mRS score  at discharge |
| --- | --- | --- | --- | --- | --- | --- |
| 1 | 47/Female | Headache | ICA | SAH | N/A | 6 |
| 2 | 52/ Female | Diplopia | ICA | SAH | N/A | 6 |
| 3 | 63/ Female | Incidental | ICA | ICH | 22 | 3 |
| 4 | 70/ Female | Dizziness | ICA | ICH | 12 | 3 |
| 5 | 59/ Female | Headache/ Dizziness | PC | SAH | N/A | 6 |
| 6 | 52/ Female | Headache/ Diplopia | ICA | ICH | 33 | 4 |
| 7 | 53/ Female | Incidental | ICA | ICH | 16 | 2 |
| 8 | 73/ Female | TIA | ICA | SAH | N/A | 4 |
| 9 | 58/ Female | Impaired vision | ICA | SAH | N/A | 2 |
| 10 | 54/ Female | Incidental | ICA | SAH | N/A | 3 |
| 11 | 54/ Female | Headache/ Dizziness | PC | SAH | N/A | 2 |

N/A, not applicable.

TIA, transient ischemic attack; ICA, internal carotid artery; PC, posterior circulation; SAH, subarachnoid hemorrhage; ICH, intracerebral hemorrhage; mRS, modified Rankin scale.

**Supplemental table 2. The univariate Cox regression for the risk related to PB**

| Characteristics | Hazard ratio | 95% CI | *P* value |
| --- | --- | --- | --- |
| Age | 1.04 | 0.99-1.10 | 0.137 |
| Male | 1.23 | 0.43-3.50 | 0.702 |
| Hypertension | 0.49 | 0.16-1.50 | 0.212 |
| Dyslipidemia | 0.59 | 0.19-1.82 | 0.362 |
| Diabetes mellitus | 0.49 | 0.16-1.5 | 0.453 |
| Coronary artery disease | 0.85 | 0.11-6.42 | 0.874 |
| Current smoker | 1.48 | 0.43-5.16 | 0.539 |
| Regular alcohol abuse | 0.04 | 0.00-61.07 | 0.396 |
| Surgical methods (FD vs. FD+coils) | 2.14 | 0.63-7.32 | 0.224 |
| SMT users | 0.12 | 0.02-0.95 | 0.045^┼^ |
| Location |  |  |  |
| ICA | Reference | | |
| MCA | 0.00 | 0.00 | 0.981 |
| AcomA/ACA/Posterior | 1.11 | 0.25-4.83 | 0.899 |
| Bifurcation | 8.72 | 3.22-23.59 | <0.001^┼^ |
| Irregular shape | 1.83 | 0.60-5.62 | 0.289 |
| Aneurysm size |  |  |  |
| <7mm | Reference | | |
| 7-10mm | 0.24 | 0.30-1.91 | 0.686 |
| >10mm | 0.82 | 0.31-2.17 | 0.247 |

^┼^, the parameter was significant.

PB, postoperative bleeding; ACA, anterior cerebral artery; AcomA, anterior communicating artery; ICA, internal carotid artery; MCA, middle cerebral artery.
